# The Peoples' Want in North London

**Published:** 1887-01-22

**Authors:** C. T. Murdoch

**Affiliations:** Chairman. The Great Northern Central Hospital, N.


### The Editor's Letter-Box.

[Our Correspondents are reminded that prolixity is a great bar to publication, and that brevity of style and conciseness of statement greatly facilitate early publication.]

### THE PEOPLE'S WANT IN NORTH LONDON.

When so much is being done in *East* London for the edification and enjoyment of the working-class, allow me to draw the attention of the wealthy and generous to the important undertaking which has just been commenced in *North* London for their benefit—that of building a Hospital for the medical wants of this populous but poor district. All will agree that sickness should have our first consideration, but in the neighbourhood of Finsbury Park, Islington, Highbury, and part of Camden and Kentish Towns there is now hardly any provision for the treatment of the sick poor. It is estimated that at least 100,000 workpeople, with many clerks and shop employés live in North London, yet the only Hospital accommodation for these and their families is that afforded by the Great Northern Central Hospital, with but thirty-two beds and a small and ill-arranged out-patient department. In fact, with a population of nearly a million and a half, there is in the district one bed only available for every 25,000 of the inhabitants, whereas competent authorities deem one for every 700 absolutely necessary.

A movement with the Baroness Burdett-Coutts and the Duke of Westminster at the head, has been started to build a Hospital of 150 beds in the centre of this thickly-populated part of the Metropolis. The work has been commenced, but funds are coming in but slowly towards the £45,000 which is required. The object, therefore, of this letter is to point out to the public that this want is most pressing, and to ask for a share of their bounty, at this benevolent season of the year. C. T. Murdoch, Chairman.

The Great Northern Central Hospital, N.

